# Optimizing Precision Medicine for Breast Cancer Brain Metastases with Functional Drug Response Assessment

**DOI:** 10.1158/2767-9764.CRC-22-0492

**Published:** 2023-06-21

**Authors:** Aki Morikawa, Jinju Li, Peter Ulintz, Xu Cheng, Athena Apfel, Dan Robinson, Alex Hopkins, Chandan Kumar-Sinha, Yi-Mi Wu, Habib Serhan, Kait Verbal, Dafydd Thomas, Daniel F. Hayes, Arul M. Chinnaiyan, Veerabhadran Baladandayuthapani, Jason Heth, Matthew B. Soellner, Sofia D. Merajver, Nathan Merrill

**Affiliations:** 1 Department of Internal Medicine, University of Michigan, Ann Arbor, Michigan.; 2 Department of Biostatistics, University of Michigan, Ann Arbor, Michigan.; 3 Department of Pathology, University of Michigan, Ann Arbor, Michigan.; 4 Department of Neurosurgery, University of Michigan, Ann Arbor, Michigan.; 5 Department of Chemistry, University of Michigan, Ann Arbor, Michigan.

## Abstract

The development of novel therapies for brain metastases is an unmet need. Brain metastases may have unique molecular features that could be explored as therapeutic targets. A better understanding of the drug sensitivity of live cells coupled to molecular analyses will lead to a rational prioritization of therapeutic candidates. We evaluated the molecular profiles of 12 breast cancer brain metastases (BCBM) and matched primary breast tumors to identify potential therapeutic targets. We established six novel patient-derived xenograft (PDX) from BCBM from patients undergoing clinically indicated surgical resection of BCBM and used the PDXs as a drug screening platform to interrogate potential molecular targets. Many of the alterations were conserved in brain metastases compared with the matched primary. We observed differential expressions in the immune-related and metabolism pathways. The PDXs from BCBM captured the potentially targetable molecular alterations in the source brain metastases tumor. The alterations in the PI3K pathway were the most predictive for drug efficacy in the PDXs. The PDXs were also treated with a panel of over 350 drugs and demonstrated high sensitivity to histone deacetylase and proteasome inhibitors. Our study revealed significant differences between the paired BCBM and primary breast tumors with the pathways involved in metabolisms and immune functions. While molecular targeted drug therapy based on genomic profiling of tumors is currently evaluated in clinical trials for patients with brain metastases, a functional precision medicine strategy may complement such an approach by expanding potential therapeutic options, even for BCBM without known targetable molecular alterations.

**Significance:**

Examining genomic alterations and differentially expressed pathways in brain metastases may inform future therapeutic strategies. This study supports genomically-guided therapy for BCBM and further investigation into incorporating real-time functional evaluation will increase confidence in efficacy estimations during drug development and predictive biomarker assessment for BCBM.

## Introduction

Breast cancer brain metastases (BCBM) are clinically challenging due to the unique features associated with this site of disease. Even small metastatic lesions in the brain often impact quality of life and mortality (1–5). The organ-specific features, such as the blood–brain barrier or the blood–tumor barrier and the highly specialized and unique tumor microenvironment, limit anticancer drug exposure (6). Currently, anti-HER2 targeted drugs are the main class of drugs used for BCBM therapy. There is a significant gap in available therapeutics against BCBM, specifically for tumors without the HER2 biomarker or resistant to anti-HER2 therapy. To fill this critical gap, several genomic and gene expression analyses have reported potential driver genes or oncogenic pathways associated with BCBM and motivated the expansion of the precision medicine approach to patients with brain metastases (7–10). Currently ongoing clinical trials, such as the American Society of Clinical Oncology's TAPUR: Testing the Use of FDA Approved Drugs That Target a Specific Abnormality in a Tumor Gene in People with Advanced Stage Cancer (NCT02693535) and Alliance's A071701: Genetic Testing in Guiding Treatment for Patients with Brain Metastases (NCT03994796), provide an important opportunity to examine the use of therapies believed to be “genomically-matched” against brain metastases (11, 12).

There are several FDA-approved targeted drugs such as tucatinib and osimertinib that have been active against brain metastases (13, 14). As these drugs are not available under the FDA label to many patients with brain metastases, precision medicine aims to expand therapeutic options for tumors without a validated molecular target or which have progressed on approved regimens. While these early-stage precision medicine approaches are actively being examined in patients with brain metastases, the observations and results from such genomically-matched trials have led to mixed, sometimes disappointing results in terms of clinical outcomes when using a single molecular alteration (largely DNA/mutation-based) as a putative predictive biomarker for molecularly targeted therapy selection (15). For example, a review of three genomically-matched trials which screened over 13,000 patients found an overall response rate of 7.5% (16). There is currently a critical knowledge gap in how to refine and operationalize the notion of precision medicine for cancer beyond the very few established clinical biomarkers (ER, Her2) and current genomic “matching” strategies.

As the metastatic process involves a complex network of often interconnected biological pathways and cellular processes, we hypothesized that examining genomic change contextually and agnostically, as a set of related genes, may provide additional insight into BCBM. Furthermore, the validation of a bench platform that enables the testing *ex vivo* of patient material would prove an additional integrated dimension to facilitate personalized approaches. For this purpose, we propose that a functional drug screening platform with a preclinical model that reflects the complex heterogeneity of metastases may compliment current approaches that heavily rely on a static predictive biomarker, often based on a single molecular alteration detected at one timepoint. In this study, we conducted a multilevel integrative approach to molecularly profile and analyze BCBM. To test the functional significance of molecular alterations in the brain metastases as drug targets, we correlated the genomic profiles with high throughput drug screening of patient-derived xenografts (PDX) established from recently resected brain metastases of patients with metastatic breast cancer.

## Materials and Methods

### Collection of Archived Tissues and Associated Clinical Characterization

We searched the University of Michigan database for tissue archived from resected brain metastases and matched primary breast cancer tissue. The samples were included if a pair of brain metastases and primary breast tumor had enough available tissue in the block to be submitted for research molecular profiling, based on review by our translational pathology core team. A retrospective chart review was conducted to collect associated clinicopathologic information, including but not limited to estrogen receptor (ER), progesterone receptor (PR), and HER2 status and cancer-directed treatment. We obtained Institutional Review Board (IRB) approval at the University of Michigan (Ann Arbor, MI) to conduct this study.

### Molecular Profiling of Clinical Tumor Specimens

Integrative molecular sequencing (exome sequencing and whole transcriptome) analysis on formalin-fixed paraffin-embedded tissues was conducted by the overall method developed and previously published by the University of Michigan Oncoseq (MI-ONCOSEQ) program (17). The archived tumor and normal tissue samples were reviewed by a pathologist to identify areas for sequencing that contained tumor tissue. The nucleic acid isolation, library preparation, and RNA integrity check for the integrative sequencing (DNA and RNA) methods were performed according to the previously published protocol (17), with the following exceptions: paired-end reads were aligned to the hg38 (GRCh38) human reference genome rather than hg19, and variant calling was performed via Sentieon DNAScope and TNScope workflows rather than VarScan2. Briefly, 40–50 M paired end Illumina reads were generated per sample and sequenced on an Illumina HiSeq2500. In addition to standard alignment and variant calling, tumor purity was assessed using an internally developed method; copy-number aberrations were quantified and reported for each gene as the segmented normalized log_2_-transformed exon coverage ratios between each matched tumor-normal sample. Strand-specific RNA sequencing (RNA-seq) libraries were analyzed using the CRISP clinical RNA-seq pipeline which comprises expression analysis, virus detection, and structural variant detection was conducted using CODAC.

### Establishment of PDXs

Surgically resected brain metastases tissue from the University of Michigan Central Nervous System banking program was obtained fresh from the operating room. For implantation, the tumor pieces were cut into smaller fragments (approximately 1–2 mm in diameter), mixed with matrigel, and injected into the mammary fat pad of female NOD/scid/IL2R (NSG) mice as published previously (18). Resected tumors were dissociated using the Tumor Dissociation Kit (Miltenyi Biotec # 130-095-929). Mouse cells were removed using the Mouse Cell Depletion Kit (Miltenyi Biotec # 130-104-694), and DNA was collected from each PDX model using the Qiagen AllPrep mini kit (Qiagen, 80204; ref. 18). The retrospective chart review was conducted to collect the clinicopathologic information associated with the source tumor. The collection of tumor bank tissue and animal use were conducted under the protocols approved by the IRB and the Institutional Animal Care and Use Committee, respectively, at the University of Michigan (Ann Arbor, MI).

### PDX DNA Sequencing

Sequencing libraries were prepared from 100 to 200 ng of PDX-derived DNA using NEBNext Ultra II FS DNA Library Prep Kit for Illumina (New England Biolabs) per manufacturer's protocol. Libraries were checked for quantity by Qubit dsDNA High Sensitivity Assay (Thermo Fisher Scientific), and quality by TapeStation (Agilent), and pooled for capture as 2–16 samples per pool. Each pool underwent exome capture using the IDT xGen Exome Research Panel v1.0 using manufacturer's protocols (Integrated DNA Technologies), an assay with a 34 Mb target region (39 Mb of total probe length). A total of 100–300 ng of library was pooled and hybridized to the xGen Exome Panel v1.0 for 4–16 hours, and washed/captured DNA was recovered using Streptavidin beads. Purified, captured DNA was then amplified with six to eight cycles PCR. Final capture pools were checked for quality and quantity by TapeStation (Agilent), and a MiSeq sequencing run was performed prior to full sequencing to verify sample load balance. Libraries were sequenced on an Illumina NovaSeq S4 per manufacturer's protocols (Illumina) using paired-end 150 bp cycle runs. These libraries were sequenced as part of two multiplexed cohorts with targeted coverage depths of 290x (six of the seven PDX samples), and 252x (PDXBC9).

Raw sequence data were demultiplexed and converted to FASTQ files for analysis. For each sample, the *xengsort* xenograft sorting algorithm was used to discriminate reads matching the human GRCh38 genome versus the mouse GRCm39 genome, retaining only human-only reads for downstream processing (19). Overall, data were analyzed using the Sarek nf-core germline Nextflow workflow (https://nf-co.re/sarek) version 2.7, implementing Broad Institute “Best Practice” single-sample standards (20, 21)). Briefly, reads were adapter-trimmed using TrimGalore v0.6.4_dev and aligned to the human GRCh38 reference genome using the Burrows-Wheeler aligner. Duplicates were marked and reads recalibrated using GATK v4.1.7 tools, and variants called using HaplotypeCaller. MultiQC v.1.8 and QualiMap v2.2.2_dev tools were used for quality control (QC) profiling of raw and aligned reads. Variant calls were annotated with SnpEff v4.3. Resulting VCF files from the Sarek workflow were further flagged and filtered using bcftools, retaining only PASS variants after applying standard threshold flags using GATK *VariantFiltration* (SNP flags: FS > 60.0, MQ < 40.0, MQRankSum < −12.5, QD < 2.0, QUAL < 30.0, SOR > 3; INDEL flags: QC < 2.0, Qual < 30.0, FS > 200.0, ReadPosRankSum < −20.0). Resulting VCF files were annotated and assembled using the VarSeq software (GoldenHelix), mapping variants to RefSeq gene models (v. 109, 20201120), as well as adding impact prediction score voting from dbNSFP, ClinVar annotations (12/02/2021), and gnomAD population frequencies (v2.0.1). Variants with fewer than 4 reads supporting the alternate allele, and alternate allele frequencies less than 0.01, were removed via the VarSeq filter chain. For comparison with the MiOncoSeq-generated (somatic) variant calls, the target region was restricted to that of the MiOncoSeq assay, and variant calls were further filtered by removing variants those identified via MiOncoSeq germline variant calling for the normal sample matched to the primary tumor of each patient. As one final annotation, variants mapping to genes identified as having actionable drug associations in the OncoKB database were marked (22).

Quality of the raw FASTQ RNA reads data for each sample were checked using FastQC (version v0.11.3) to identify features of the data that may indicate quality problems (e.g., low-quality scores, overrepresented sequences, inappropriate GC content). Reads were adapter-trimmed using cutadapt 1.8.1. Reads were aligned to the reference genome (human hg38/GRCh38) using TopHat (version 2.0.13) and Bowtie2 version 2.2.1.0. Default parameter settings were used for alignment. A second round of FASTQC quality control (postalignment) was run to validate adapter removal.

### Differential Gene Expression and Gene Set Enrichment Analysis

Read counts from RNA-seq were used for differential gene analysis performed by DESeq2 (23). Genes were considered significantly changed with absolute log_2_ fold change > 0.6 and FDR-adjusted *P* < 0.05. Gene set enrichment were performed using gene set enrichment analysis (GSEA), gene set variation analysis (GSVA), and iPathwayGuide (24). In GSEA (GSEAv4.0.1) analysis, Hallmark, Oncogenic, Kyoto Encyclopedia of Gene and Genomes (KEGG), and Gene Ontology Biological Process (GoBp) gene sets of v7.4 from MSigDB were used to identify significantly upregulated or downregulated signaling pathways (25, 26). A total of 10,000 permutations were performed for weighting for scores of genes ranking. Gene sets with FDR-adjusted *P* < 0.05 was considered as significant changes (27). RNA-seq reading counts normalized by transcript per million (TPM) were used for performing GSVA (GSVAv1.40.1). The same gene sets used for GSEA were selected for the corresponding GSVA. The output of GSVA is a matrix containing pathway enrichment scores for each gene set and each sample (28). Furthermore, the iPathwayGuide (Advaita Bio's iPathwayGuide) analysis was performed for the significantly impacted pathways with inputting of significantly changed genes with both upregulated and downregulated directions from DESeq2. Overrepresentation analysis was used to determine the predefined gene sets that were significantly changed across brain metastases and primary breast tumor groups (29).The KEGG gene set category was selected for iPathway analysis (24).

### Drug Testing

Cells were screened in 384-well ultra-low attachment plates (Corning, 4516 or S-Bio, MS-9384WZ) in singlicate or duplicate, 7-point dose–response format. Cells were plated in our previously defined SM+6 medium (30). Cells were plated on day 0 at 3,000 cells per well. On day 0, drugs were added at 1:1,000 using a 50 nL pin tool, resulting in 0.1% final DMSO concentration per well. On day 5, viability was measured using CellTiter-Glo 3D (Promega, G9683) on an Envision plate reader (Perkin Elmer). Cell viability was calculated as %viability or %inhibition with respect to vehicle. Data were archived and analyzed using the CDD Vault from Collaborative Drug Discovery (www.collaborativedrug.com). Drug sensitivity score 3 (DSS_3_) values were calculated as described by Yadav and colleagues (31). IC_50_ value, hillslope, maximum inhibition, and drug range were entered into the DSS package for Rstudio and DSS_3_ values (ranging 0 to 100) were calculated.

### Data Availability Statement

The data generated in this study are available within the article and its Supplementary Data. Other data generated in this study are available upon reasonable request to the corresponding author.

## Results

### Case Characteristics

Of the patients with BCBM who had initial primary breast tumor surgery and subsequent brain metastases resection at the University of Michigan, 12 pairs were identified: Group A: BCBM series (Supplementary Fig. S1a). Archived samples were collected from 1994 to 2018. Five of 12 (42%) cases were HER2 positive. The median age at the time of brain metastases surgery was 50 years old (range: 27–67). All patients had been exposed to cytotoxic chemotherapy prior to the brain metastases sample collection. The brain metastases analyzed were from resections as the initial brain-directed therapy, except for one case (BCBM001), for whom the resection was done for a progression in the brain after whole-brain radiotherapy. The patient characteristics including the receptor biomarker information are summarized in Supplementary Fig. S1b.

### Gene-specific Analysis

Most of the potentially significant alterations, including all *TP53* mutations from fully characterized pairs, were conserved between brain metastases and the matched primary breast tissue (Supplementary Fig. S2). The integrated analysis of gene-specific evaluation observed that *TP53* mutation was the most common alteration noted in this case series, regardless of clinical subtype (11 of 12 cases)**.** Additional potential driver alterations noted in the brain metastases (but not presented in the matched primary breast tumor) included *CDK2NA* loss in addition to *PI3KCA* and *ESR1* mutations.

BCBM011 was a case with additional sequencing analysis from lung metastases (Supplementary Fig. S3). Both the lung and brain metastatic lesions were hypermutated, showing APOBEC signature (32) The alterations noted in these samples were overall similar between the brain and the lung metastases, with the exception of *SETD1B* (p.Q371*), which was present in the brain metastases and the matched primary breast tumor, but not in the lung metastases. Conversely, the brain and lung metastases shared the hotspot *PIK3CA* E545K mutation as well as all three *ESR1* mutations (E380Q, D273N, D351H), which were not present in the matched primary breast tumor.

### Differentially Expressed Pathway Analysis

**FIGURE 1:**
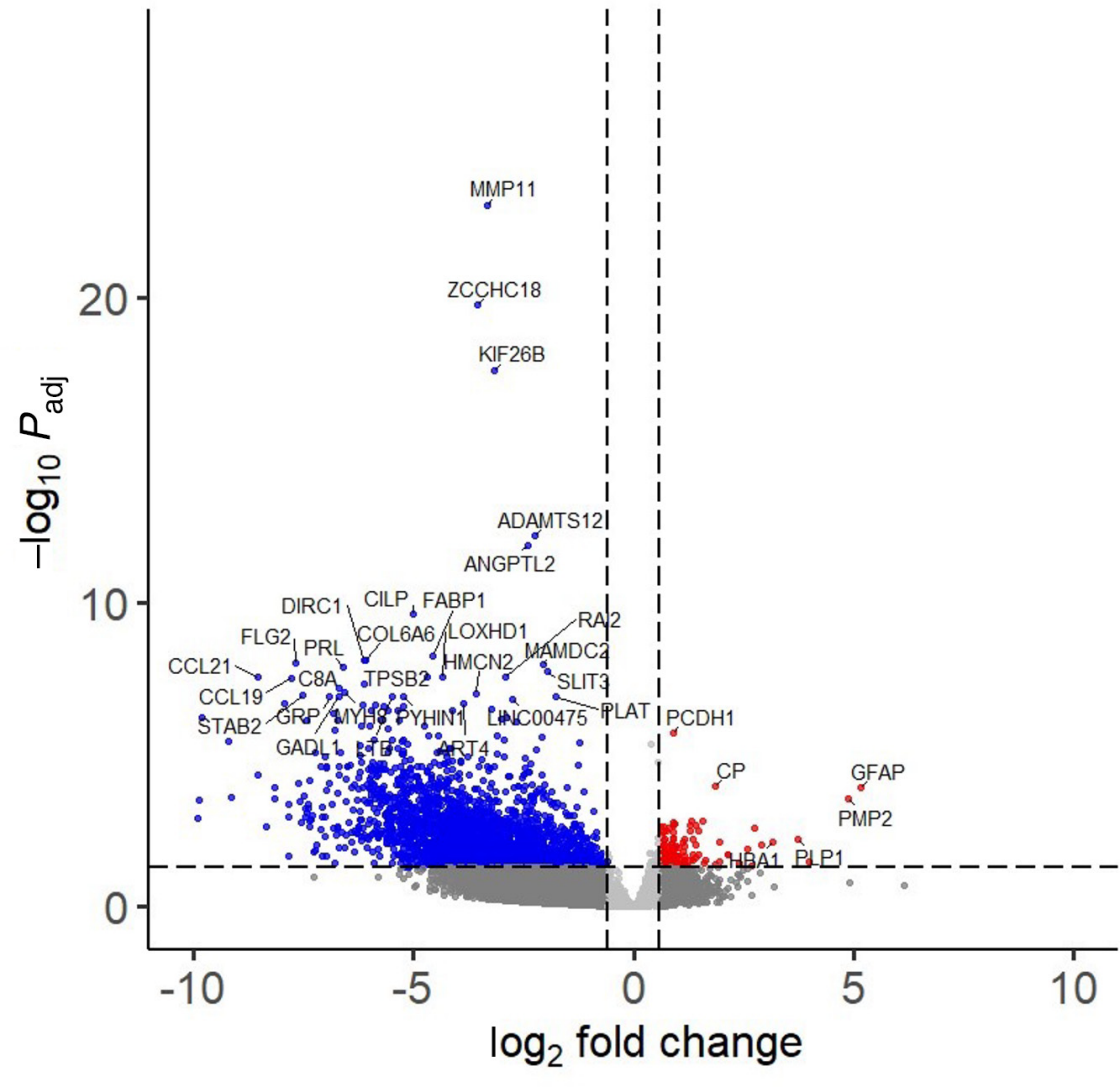
Volcano plot of differentially expressed genes comparing the paired brain metastases and primary breast tumor (Group A). The downregulated and the upregulated genes in the brain metastases samples compared with the matched primary breast tumor samples are color coded as blue (down) and red (up).

Pathway analyses were conducted to compare gene expression between the paired brain metastases and primary tumors. Eight of 12 (67%) archival samples passed quality control to enable the paired sample gene expression analysis. Overall, there were more downregulated genes in brain metastases compared with paired primary breast tumors, as shown in Fig. 1. A total of 149 genes were upregulated compared with 2,751 genes downregulated in the brain lesions. There were 56 differentially regulated signaling pathways identified using iPathway analysis. These included olfactory transduction, primary immunodeficiency, PI3K-AKT signaling, tyrosine metabolism, chemokine signaling, JAK-STAT signaling, basal cell carcinoma, T-cell receptor signaling, Wnt signaling, EGFR tyrosine inhibitor resistance, RAS signaling, and breast cancer signaling. The top three differentially affected pathways were olfactory transduction, primary immunodeficiency, and protein digestion and absorption. Olfactory transduction was the top differentially expressed pathway and included several odorant receptor genes (Supplementary Fig. S4), as would be potentially expected in comparisons of brain tissue with any other tissue, due to the prominent expression of this large family of genes in brain.

**FIGURE 2:**
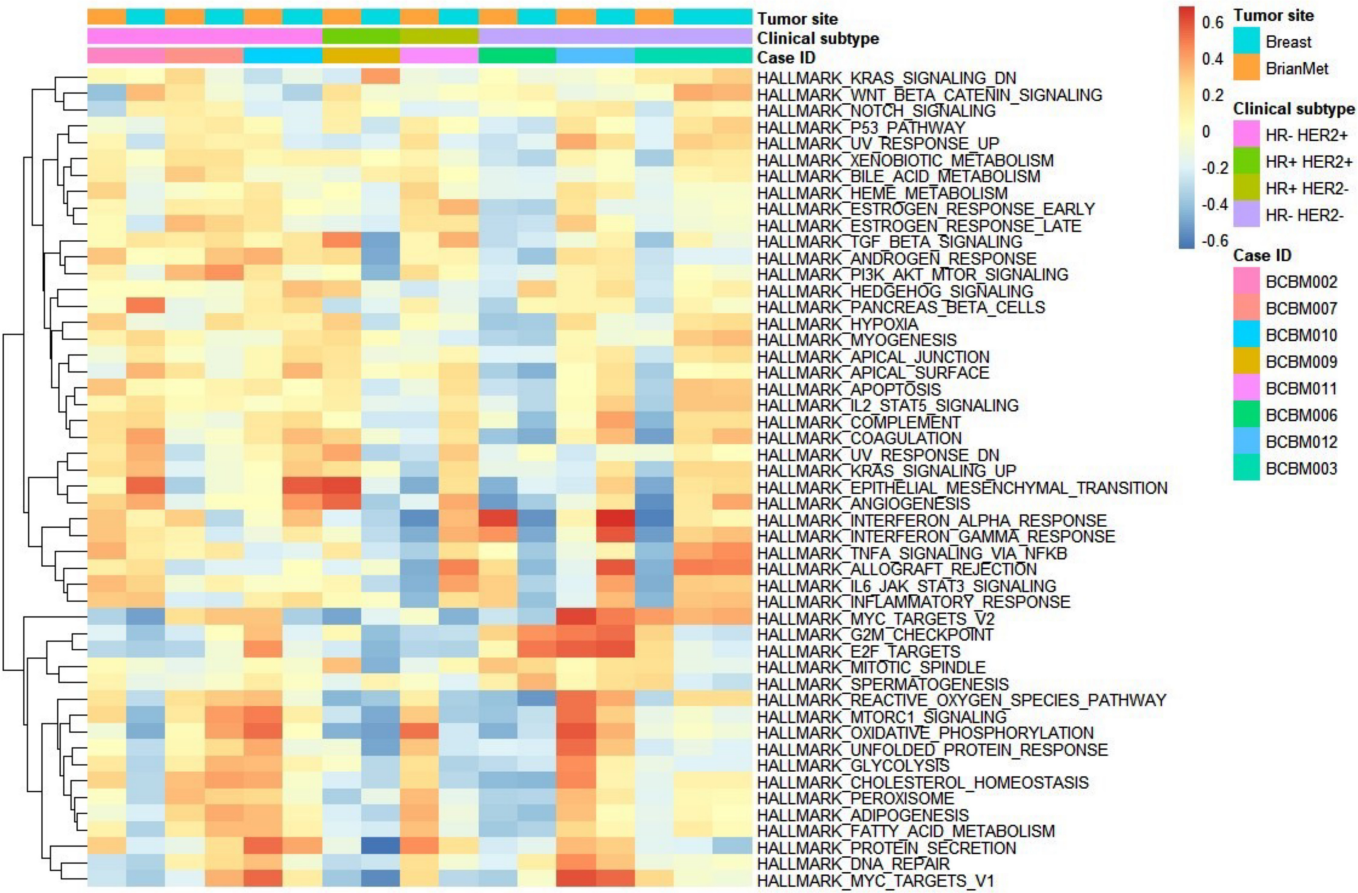
Heat map from the Hallmark gene sets GSVA comparing the paired brain metastases and primary breast tumor (Group A). HR, hormone receptor; HER2, human epidermal growth factor 2.

The GSEA revealed sets of enriched and depleted genes. From the hallmark gene set, there were five and nine gene sets observed to be significantly enriched and depleted at FDR < 0.05, respectively (Table 1). Interestingly, protein secretion, oxidative phosphorylation, and adipogenesis, in addition to androgen response, mitotic spindle, and *myc*-related sets were also enriched. In the depleted set, a *KRAS*-related set was noted, as well as immune and inflammatory-related sets. To complement the GSEAs, we also conducted a patient-specific GSVA of the Hallmark gene sets and the corresponding heat map for the paired samples is shown in Fig 2. We also examined oncogenic gene sets. Of the enriched gene sets, as previously noted by others (33, 34), growth factor pathways such as *ERBB2* and *EGFR,* as well as *MTOR* and *AKT-*related sets were enriched. Of the depleted sets, the *KRAS*-related set was noted to be significantly depleted, as well as *PTEN*-related sets (Supplementary Fig. S5). Additional analyses were conducted using GSEA and significantly altered gene sets, using KEGG and GoBp, were identified (Supplementary Fig. S5; refs. 25, 26).

**TABLE 1:** GSEA using the Hallmark gene sets comparing the paired brain metastases and primary breast tumors. Gene sets with FDR < 0.05

NAME	SIZE	ES	NES	p-val	FDR q-value
**Upregulated gene sets**					
HALLMARK_PROTEIN_SECRETION	94	0.43057767	2.1916907	0	4.00E-04
HALLMARK_OXIDATIVE_PHOSPHORYLATION	199	0.31743	1.8286253	0	0.007600902
HALLMARK_MTORC1_SIGNALING	199	0.30052698	1.7314858	0	0.01158934
HALLMARK_ANDROGEN_RESPONSE	98	0.33145055	1.716175	8.02E-04	0.009625576
HALLMARK_MITOTIC_SPINDLE	199	0.2636196	1.5251341	0.001647446	0.03683615
**Downregulated gene sets**					
HALLMARK_ALLOGRAFT_REJECTION	167	−0.4972723	−2.4051008	0	0
HALLMARK_EPITHELIAL_MESENCHYMAL_TRANSITION	194	−0.4094371	−2.0216177	0	3.57E-04
HALLMARK_KRAS_SIGNALING_UP	178	−0.3900975	−1.9043354	0	0.001021529
HALLMARK_IL2_STAT5_ SIGNALING	187	−0.38098702	−1.8678038	0	0.001213369
HALLMARK_HEDGEHOG_SIGNALING	33	−0.472654	−1.70284	0.006790403	0.007752952
HALLMARK_COAGULATION	103	−0.37336287	−1.6854488	7.97E-04	0.00773936
HALLMARK_APICAL_JUNCTION	184	−0.34352946	−1.6789134	4.89E-04	0.00701823
HALLMARK_INFLAMMATORY_RESPONSE	170	−0.33855027	−1.641233	3.69E-04	0.009221255
HALLMARK_COMPLEMENT	174	−0.31858927	−1.5492525	0.002094628	0.022641048

#### PDXs from Brain Metastases

To examine whether the putatively actionable genomic alterations in brain metastases are functional drug targets, we prospectively collected fresh surgically resected BBM and established PDX models for functional assessment of drug response (Group B: PDX BC series). We selected six cases to capture three clinical subtypes of breast cancer: ER^+^HER2^−^, ER/PR/HER2^−^ [triple-negative breast cancer (TNBC)], and HER2^+^ (Supplementary Fig. S6). We compared the genomic profiling of PDXs and paired brain metastases tumors utilizing PDX samples from passage 2–3 (Supplementary Fig. S7).

Across all the samples, we noted alterations in the DNA damage repair pathway involving tumor suppressor genes such as BRCA1/2 and CHEK1/2 (Supplementary Fig. S7). Of note, two cases, PDXBC3 and PDXBC9, were known to have germline mutations in the DNA damage repair pathways BRCA1 and RAD50, but other cases with DNA damage repair alterations had no known germline mutations identified by clinical germline genetic panel testing. Two samplings from a large solitary resected tumor for PDXBC9 were available for sequencing, and the result from both samples were included in this analysis.

**FIGURE 3:**
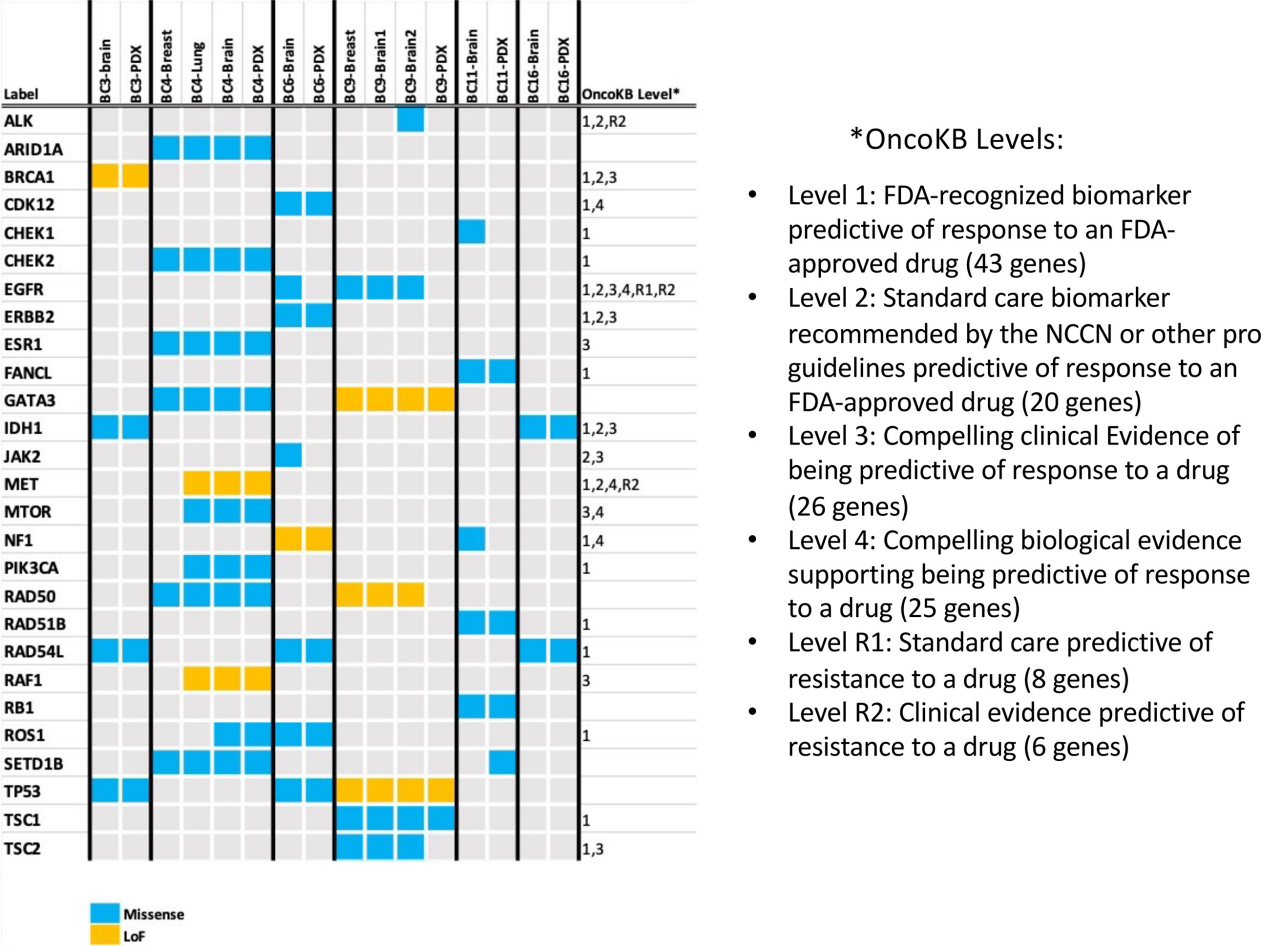
Comparisons of potentially significant molecular alterations observed in the paired clinical tumor tissue samples and the PDX models. In addition to the paired resected brain metastases and PDX samples, the molecular alterations observed in other disease sites (primary and distant metastases) are listed when the archived samples were available for sequencing. When applicable, the OncoKB levels are noted to indicate the level of evidence currently available for targeted drug therapies.

We narrowed the list of alterations down to the potentially actionable gene set by cross-matching with OncoKB, a curated database for potentially actionable genomic alterations in cancer for precision medicine (ref. 22; Fig. 3). In addition to the DNA-repair pathway genes for which a PARP inhibitor was matched, we observed alterations in ALK, ROS1, and TSC1 for which OnkoKB level 1 notation matches an FDA-approved biomarker with an FDA-approved drug. Potentially actionable targets not traditionally associated with brain metastases in breast cancer, were thus identified in all six models.

#### Genomically-directed Drug Testing

We selected PDX models with potentially actionable alterations to serve as avatars to simulate precision medicine in genomically-matched trials. We conducted drug screening *ex vivo* using a panel of over 350 drugs. We identified multiple prioritized targeted drugs in each sample, as there were multiple prioritized alterations, including some novel alterations, with no previously known role as driver mutations in breast cancer. In addition to FDA-approved targeted drugs, the drug panel included off-label and investigational drugs targeting matched alterations. Chemotherapies commonly used in breast cancer were also included in our drug screening panel (Supplementary Fig. S8). Table 2 summarizes the responses to prioritized targeted therapies against their corresponding matched targets. DSS_3_ were calculated for drug response to allow comparisons of responses across drugs with different potency and efficacy (31). Yellow highlighted numbers indicate DSS_3_ categorized as semiactive (21–29) to very active (>59). PDXBC4 (ER^+^HER2^−^) was identified as having multiple potential genomic targets with observed favorable sensitivity scoring.

**TABLE 2:**
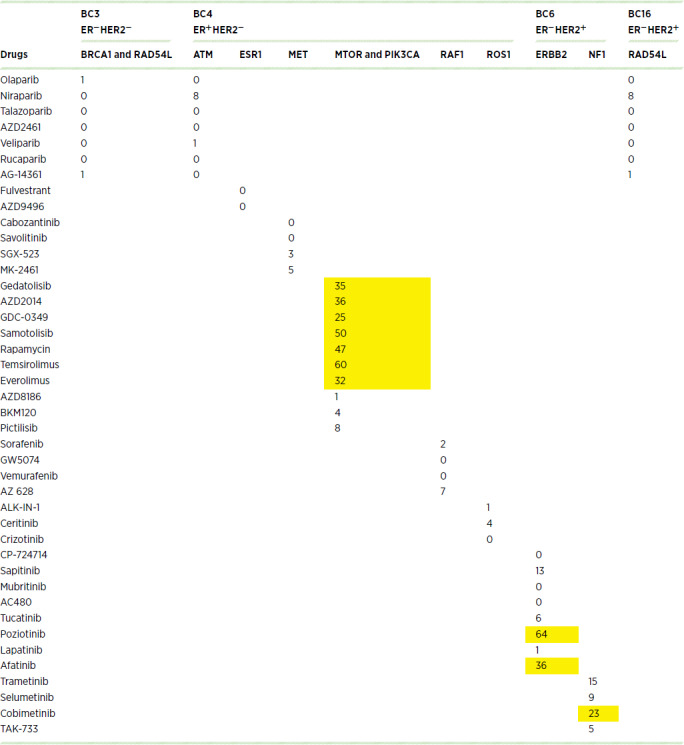
Drug responses (DSS_3_ values) matched to potentially actionable genomic alterations in BCBM PDX models

We observed that the genomic alteration-to-drug pairing was concordant for a number of drugs, but not all. For instance, fulvestrant or selective ER degraders are often recommended for ESR1-mutated cancers because ESR1 mutation is considered a marker of resistance to aromatase inhibitors (AI). In this case, the source patient for PDXBC4 (ER^+^HER2^−^) had previously progressed on AI, fulvestrant, and AI combined with a CDK4/6 inhibitor. Therefore, the low drug efficacy to fulvestrant (as defined by a low DSS_3_) is consistent with the clinical background of this PDX model. In the cases of RAF1 and ROS1 loss of function or missense mutations, drugs targeting RAF and ROS were inactive as single agents in our screen. PARP inhibitors, which may require longer exposure than our experiment allowed for, were inactive across all screened PDXs. The presence of mutations in the PI3K/AKT/mTOR pathway was overall the most successful characteristic contributing to a matching for potentially actionable mutations, with an activity range of 1 to 60 DSS_3_ values.

#### Target-agnostic Drug Testing

We conducted additional drug screening of these PDX models against a curated panel of common drugs used in breast cancer as well as drugs that demonstrate promise in other cancers. Importantly, these PDX models were established from patients who previously had exposure to chemotherapy, mostly in the early stage of disease, but some also in the metastatic setting. Consistent with the tumors’ prior chemotherapy exposures, most PDX models were resistant to anthracycline and taxane chemotherapies, commonly used in the early-stage setting, and which these patients had received. Some of the chemotherapies approved in the metastatic setting for patients with breast cancer widely differed in their average DSS_3_, depending on the specific prior exposures. In the case of PDXBC11, *ex vivo* testing suggested broad resistance to chemotherapies, including eribulin which had been used in treating only this patient, and indeed the patient had progressed on this agent prior to excision of their brain metastasis.

Next, we examined the types of molecularly targeted drugs (non-cytotoxic chemotherapy) where high DSS_3_ were observed for each PDX model, regardless of genomic alterations (Table 3). We observed several molecularly targeted drugs that were associated with high drug activity in the PDX models. For example, in BC3 (TNBC), no PI3K/AKT/mTOR mutations were noted (unknown PTEN expression status). In the BC3 PDX, Torin2 was highly active, but no mTOR-targeting drugs tested were active. Another TNBC case, PDXBC9, was associated with a high response to targeted therapies such as HSP90 and AURKA inhibitors, yet this tumor carried no known molecular alterations in the HSP90 or AURKA pathways. We previously reported bortezomib as one of the most active compounds we found during a drug screening study using 23 TNBC cell lines (35). Consistent with our previous work, both PDXBC3 and PDXBC9 had high DSS_3_ values for bortezomib. Moreover, histone deacetylase (HDAC) inhibitors, including romidepsin, another drug we reported as highly active in TNBC cell lines in our previous study, appeared to be very active across the brain metastases PDX models and not limited to TNBC.

**TABLE 3:** Molecular targeted drugs with very high DSS_3_ values

		TNBC		ER^+^HER2^−^		HER2^+^	
Drugs	Reported targets	BC3	BC9	BC4	BC11	BC6	BC16
Bortezomib	20S proteasome NFkB Induces ERK phosphorylation to suppress cathepsin B	84	61		68		68
Romidepsin	HDAC1, HDAC2, HDAC3, HDAC3, HDAC8	75		73	61	68	77
Dinaciclib	CDK1, CDK12, CDK2, CDK5, CDK9	66					70
Panobinostat	HDAC1, HDAC10, HDAC11, HDAC2, HDAC3, HDAC4, HDAC5, HDAC6, HDAC7, HDAC8, HDAC9	63		62			
YM155	BIRC5	71	83				85
ENMD-2076	AURKA, FGFR1, FGFR2, KDR, RET	66					
Torin 2	MTOR	65		60		65	
Sodium phenylbutyrate	Histone deacetylase	84	62		63		
Alisertib	AURKA		86				88
AUY922	HSP90		62				
17-DMAG	HSP90		66				66
Trametinib	MAP2K1, MAP2K2			63			
Sapanisertib	MTOR, RICTOR			66		71	
Poziotinib	EGFR, ERBB2, ERBB4					64	
Elesclomol	HSP90					75	62
AZD8055	MTORC1, MTORC2					66	60
Delanzomib	26S mammalian proteasome						63

## Discussion

Our study aimed to determine the molecular features salient or unique to brain metastatic lesions and to examine the drug efficacy profiles of genomic alterations as drug therapy targets, using a functional drug testing approach. Procurement of matched primary and metastatic breast brain metastases can be challenging, given that surgical resection is not a common procedure for brain metastases from breast cancer. Moreover, the time between the index breast tumor and the recurrence is typically years for patients with breast cancer. Hence, the number of published matched-pair analyses of resected brain metastases and primary breast tumors is sparse (10). However, when available, these intrapatient comparisons can provide valuable insights into the unique biological trajectories of cancer cells with a brain tropism and identify potential molecular vulnerabilities to target in developing novel therapeutic strategies specific to an individual patient's lesion. In our study, we found unique molecular alterations present in the brain metastases compared with the matched primary tumor in 36% (4/11) of cases in the CDK and PI3K pathways, as consistent with previously published literature (36). While the receptor status discordance has been reported, our cases had mostly concordant status based on the clinically available testing (Supplementary Fig. S1b; ref. 37). Gene expression was predominantly downregulated in the brain, but some potentially targetable genes were upregulated in the brain lesions compared with the primary tumor, and such differential expression changes can be a guide to examine potential signaling pathways to explore as targets.

In particular, we noted the prominence of cellular energy and metabolism-related pathways and the downregulation of immune and inflammatory-related pathways. There have been studies of cellular and metabolic adaptation by tumor cells to successfully establish growth in the brain microenvironment, and such metabolic adaptation provides an advantage for cancer cell outgrowth specifically in the brain compared with other metastatic sites (38–44). We noted a set of olfactory transduction genes to be significantly downregulated in the brain, somewhat unexpectedly, given the prominence of this gene family in brain tissue gene expression. As the olfactory organ is involved in immune-related function and a possible entry point into the brain for infections, we pose that the downregulation of these genes could reflect a possible shift in the immune tumor microenvironment in the setting of brain metastases (45, 46). Our work adds to a body of evidence that targeting metabolic and immune adaptations in the brain microenvironment may help interfere with the growth of brain metastases (39, 47).

In leveraging the molecular profiling information to expand treatment options, it is not infrequent that we identify multiple alterations in genes not clinically validated to be predictive biomarkers in a particular tumor type or across tumor types: this significantly limits the options considered for each patient. Moreover, the impact of tumor heterogeneity on the efficacy of molecular-matched therapies is uncertain, but it is strongly suspected of accounting for a large proportion of treatment failures, as a fraction of cells that are resistant remain alive and expand. A diverse body of evidence has reported that patient-derived preclinical models can capture the heterogeneity and genomic complexity of source tumors and predict clinical outcomes (48–51). We successfully established a valuable resource in PDXs created from the resected BCBM of patients who were undergoing clinically-indicated neurosurgical procedures. In our PDX biobanking program for brain metastases, the uptake rate of over 50% for brain metastases is higher than the rate reported for primary or other metastatic sites (50). This may reflect the aggressive nature of the brain metastatic cells (52). Unlike cell lines, PDXs are considered to capture a realistic representation of the tumor heterogeneity of the source tumor. PDXs of breast cancer are considered to reasonably represent the source tumor molecular characteristics (18). Despite the fact that different sequencing techniques were used for PDX analysis, our results captured the pathogenic and likely pathogenic genomic mutations of the paired PDX and clinical brain metastases samples.

Using PDX models as avatars, we conducted a molecular matched analysis using a tumor-agnostic panel of over 350 drugs. We found heterogeneity of concordance between genomic changes and drug sensitivities (certain alteration-drug matches worked better than others) when multiple alterations were present. Moreover, there were potentially clinically relevant differences among drugs proposed to target the same pathway, such as the PI3K/mTOR/AKT. However, all those drugs would have been listed as equivalent in next-generation sequencing (NGS) reports of tumors bearing PI3K/mTOR/AKT alterations. This is a potentially critical contribution of drug testing since genomic analyses at one timepoint essentially lists drugs that may target a given mutation, unranked. It is left to the patient and physician to make a decision from the list, without additional data on the patient's tumor. Thus, our work supports that real-time drug sensitivity testing stands to add granularity to unranked lists of drugs and begin to steer the discussion based on preliminary efficacy data of the preferred drugs, based on the patient's own tumor cells.

We also examined drugs that had the highest antitumor activity based on the DSS_3_ values without any molecular matching and found there were non-chemotherapy drugs that showed very high activity in our PDX models. These drugs would not have been nominated on the basis of molecular profiling alone. This discovery will lead to future studies examining the biology and therapeutic options, especially for chemotherapy-resistant tumors such as the case for the patient bearing tumor BC11. Our findings are consistent with other published reports and further support the incorporation of rigorous *ex vivo* functional testing in precision medicine approaches (53, 54).

Our study has some limitations. Our sample size is small overall; however, this is largely due to the feasibility of collecting matched breast primary and brain metastases tissue pairs, from time points often separated by many years or decades. Recently, a review article by Morgan and colleagues has curated published genomic profiling studies of BCBM (10). In the review, patient sample sizes with paired primary and metastases ranged from 0 to 21 patients, with a median of 10 patients across 12 published studies cited. Therefore, relative to the literature, our sample size is reflective of this inherent challenge of collections of the brain metastatic site. Despite this unavoidable limitation given sample availability as a limiting variable, our study contributes a valuable set of data for understanding the molecular landscape of BCBM and establishes new BCBM PDX models, including the relatively rare ER^+^HER2^−^ clinical subtype (55, 56). Moreover, these PDX models can be further utilized to interrogate the mechanisms underlying the target engagement and resistance associated with these molecularly targeted drugs.

In our study, the PDX models retained alterations that are considered “potentially” clinically actionable using the OnkoKB database. However, some discordances were noted between the source and PDX pairs as well. There have been recent reports of modest molecular divergence even in early passage PDX samples (50), which may provide an explanation for the observed divergences. Spatial heterogeneity present in the original tumor may also play a role in divergence from tumor lesion to PDX. When not limited to the OnkoKB database matches to curated alterations, the rate of discordance was low among all the genomic alterations noted in our samples. It is unclear whether the drug sensitivity would have been impacted by these noted differences. Knowledge about the clinical utility of PDX models in predicting drug sensitivity will be advanced by ongoing prospective clinical trials that are incorporating the use of PDX models (48, 49, 57). Exploring the impact of passages on drug sensitivity in PDX models is also an area of future investigation in our ongoing brain metastases PDX characterization effort. As with all prior PDX studies, immune oncology drugs were not included in our studies as an intact human immune system is required for proper testing. We are developing *in vitro* technologies to help overcome this limitation.

We also recognize that our drug testing approach using PDXs warrants further *in vivo* validation which is currently ongoing. The need for corroborating patient data is a limitation that requires future investigation to translate our proposed approach to the clinic. Considering the current study evaluated non-standard therapies with a limited representation of the ER^+^ HER2^−^ subtype, prospective clinical validation of our approach is strongly desired. However, on the other hand, it is important to recognize that the time required to establish a PDX may make the validation clinically challenging, as many patients with brain metastases can succumb to the disease quite rapidly; thus, this patient population presents the urgent clinical need of selecting potentially effective treatment options within a shorter time frame than PDX generation allows. To address the timeliness of informative results, we are currently conducting a real-time drug testing of patient-derived organoids (PDO) directly established from the freshly resected surgical specimen from patients with BCBM with a personalized set of drugs (58). If successful, the use of PDOs would considerably shorten the time to drug testing, be less cost-prohibitive, and may decrease the likelihood of molecular divergence of the source tumor.

In summary, our data lend strong support to the use of molecular targeted therapies in BCBM and further investigation of the complementary use of functional testing to enrich the precision oncology information that can be provided to the patient and provider, adding to the current genomic profiling practice. By utilizing a large tumor-agnostic panel of over 350 drugs, we found unexpected and hypothesis-generating drug activities across our PDX models that would not have been on lists generated from NGS alone. The patient-derived preclinical models are rare for BCBM. The brain metastases PDX models developed in this study are valuable tools to further examine drug sensitivity and resistance mechanisms. Moreover, these models are well suited to interrogate the biology of the disease process in the clinically-relevant background of genomically complex and heterogenous tumors. On the basis of our work, we propose that there is a future need to further refine the incorporation of functional testing to complement the molecular profiling for brain metastases in a clinically-informative approach.

## Supplementary Material

Supplementary Data S1a and S1b S1a. Breast cancer brain metastases cohort (Group A) selection S1b. Clinical characteristics of breast cancer brain metastases cohort (Group A)

Supplementary Data S2 Molecular aberrations comparing paired primary breast cancer and brain metastases: Group A case series.

Supplementary Data S3a and S3b Genomic comparison across the different metastatic sites compared to the matched primary. A. Genomic alterations across metastatic sites compared to matched primary B. Copy number variations across metastatic sites compared to matched primary

Supplementary Data S4 Ipathway analysis table

Supplementary Data S5 GSEA analysis using the oncogenic, KEGG, and GOBP gene sets.

Supplementary Data S6 Clinicopathologic characteristics of source patients for brain metastases PDX models: Group B.

Supplementary Data S7 PDX molecular profiling

Supplementary Data S8 DSS3 values for chemotherapies commonly used in breast cancer treatment
