# Supplementary material for: Optimizing Precision Medicine for Breast Cancer Brain Metastases with Functional Drug Response Assessment
Source: Cancer Res Commun. 2023 Jun 21;3(6):1093–103. doi: 10.1158/2767-9764.CRC-22-0492 (PMC10284082; doi:10.1158/2767-9764.CRC-22-0492)
Supplement: Supplementary Data S1a and S1b — S1a. Breast cancer brain metastases cohort (Group A) selection S1b. Clinical characteristics of breast cancer brain metastases cohort (Group A) [file crc-22-0492-s01.docx]

**S1a. Breast cancer brain metastases cohort (Group A) selection**

**
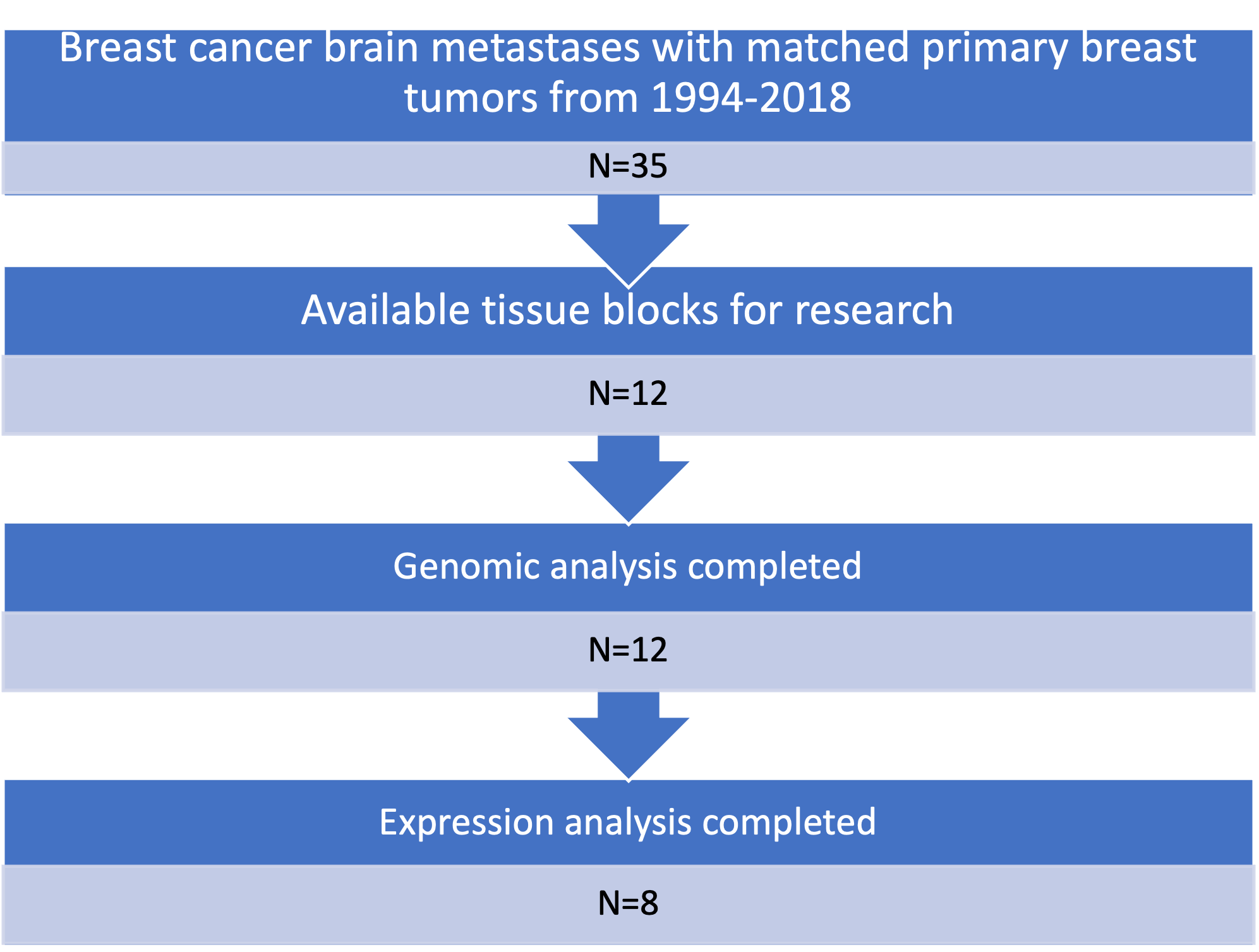
**

**S1b. Clinical characteristics of breast cancer brain metastases cohort (Group A)**
