# Supplementary material for: Optimizing Precision Medicine for Breast Cancer Brain Metastases with Functional Drug Response Assessment
Source: Cancer Res Commun. 2023 Jun 21;3(6):1093–103. doi: 10.1158/2767-9764.CRC-22-0492 (PMC10284082; doi:10.1158/2767-9764.CRC-22-0492)
Supplement: Supplementary Data S2 — Molecular aberrations comparing paired primary breast cancer and brain metastases: Group A case series. [file crc-22-0492-s02.pdf]

S2. Molecular aberrations comparing paired primary breast cancer and brain metastases: Group A case series.

Case ID	Clinical subtype	Breast primary	Brain met
**BCBM001**	TNBC	TP53 (L257P)	TP53 (L257P)
**BCBM002**	HER2+	TP53 (R273H)	TP53 (R273H)
**BCBM003**	TNBC	TP53 (R248L)	TP53 (R248L)
**BCBM004**	HER2+	TP53 (R273C)	TP53 (R273C)
**BCBM005**	TNBC	Insufficient data due to poor coverage	TP53 (S106_R110 del)<br>PIK3R1 (K567E), LOH
**BCBM006**	TNBC	TP53 (C176S), LOH	TP53 (C176S), LOH
**BCBM007**	HER2+	TP53 (R248Q), LOH	TP53 (R248Q), LOH<br>***CCNE1 Amplification***
**BCBM008**	TNBC	TP53 (Q104*), LOH<br>NOTCH2 (Y2392*)<br>PTEN deletion<br>***CDH1 deletion***	TP53 (p.Q104*), LOH<br>NOTCH2 (p.Y2392*)<br>PTEN deletion
**BCBM009**	HER2+	TP53 (V173L), LOH<br>CCND1 Amplification	TP53 (V173L), LOH<br>CCND1 Amplification<br>***CDKN2A deletion***
**BCBM010**	HER2+	TP53 (G244D)	TP53 (G244D)
**BCBM011**	HR+HER2-	ARID1A (Q1095fs)<br>GATA3 (V314fs, G378fs)<br>SETD1B (Q371*)	ARID1A (p.Q1095fs)<br>GATA3 (p.V314fs, p.G378fs)<br>SETD1B (p.Q371*)<br>***Hyper-mutation (APOBEC)***<br>***PIK3CA (E545K)***<br>***ESR1 (E380Q, D273N, D351H)***
**BCBM012**	TNBC	RB1 (Splice acceptor), LOH<br>TP53 (S90fs), LOH	RB1 (Splice acceptor), LOH<br>TP53 (p.S90fs, p.W91fs), LOH
