# Supplementary material for: Optimizing Precision Medicine for Breast Cancer Brain Metastases with Functional Drug Response Assessment
Source: Cancer Res Commun. 2023 Jun 21;3(6):1093–103. doi: 10.1158/2767-9764.CRC-22-0492 (PMC10284082; doi:10.1158/2767-9764.CRC-22-0492)

S3. Genomic comparison across the different metastatic sites compared to the matched primary. A. Genomic alterations across metastatic sites compared to matched primary B. Copy number variations across metastatic sites compared to matched primary

A.

Breast primary	Brain met	Lung met
**TMB 5**	TMB 28.7	TMB 35.7
ARID1A (Q1095fs)	ARID1A (p.Q1095fs)	*ARID1A (p.Q1095fs)*
GATA3 (V314fs, G378fs)	GATA3 (p.V314fs, p.G378fs)	*GATA3 (p.V314fs, p.G378fs)*
SETD1B (Q371*)	SETD1B (p.Q371*)	
	Hyper-mutation (APOBEC)	*Hyper-mutation (APOBEC)*
	PIK3CA (E545K)	*PIK3CA (E545K)*
	ESR1 (E380Q, D273N, D351H)	*ESR1 (E380Q, D273N, D351H)*

B.

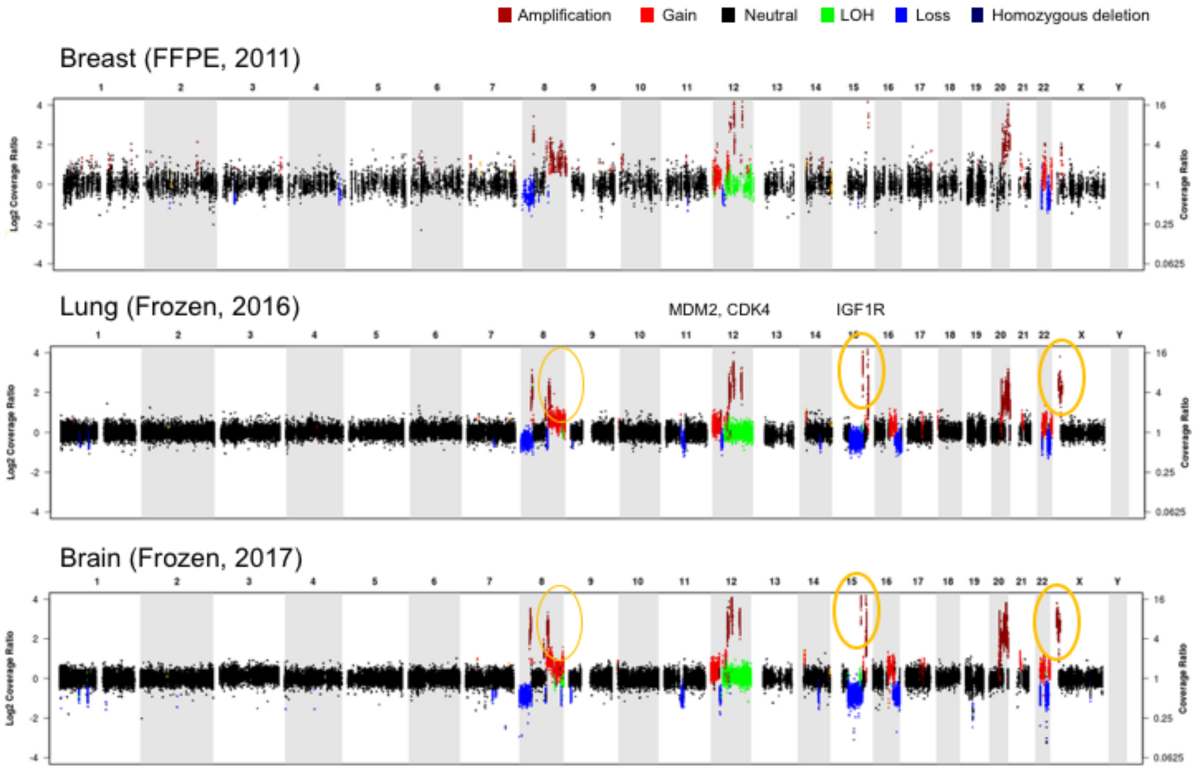

Supplement: Supplementary Data S3a and S3b — Genomic comparison across the different metastatic sites compared to the matched primary. A. Genomic alterations across metastatic sites compared to matched primary B. Copy number variations across metastatic sites compared to matched primary [file crc-22-0492-s03.pdf]
