# Supplementary material for: Optimizing Precision Medicine for Breast Cancer Brain Metastases with Functional Drug Response Assessment
Source: Cancer Res Commun. 2023 Jun 21;3(6):1093–103. doi: 10.1158/2767-9764.CRC-22-0492 (PMC10284082; doi:10.1158/2767-9764.CRC-22-0492)
Supplement: Supplementary Data S4 — Ipathway analysis table [file crc-22-0492-s04.pdf]

S4. Ipathway analysis table

Pathway name	Pathway Id	p-value	p-value (FDR)	p-value (Bonferroni)
Olfactory transduction	04740	7.478e-7	2.311e-4	2.311e-4
Primary immunodeficiency *	05340	7.754e-6	5.245e-4	0.002
Protein digestion and absorption *	04974	7.754e-6	5.245e-4	0.002
Hematopoietic cell lineage *	04640	7.754e-6	5.245e-4	0.002
Cytokine-cytokine receptor interaction	04060	8.488e-6	5.245e-4	0.003

* the p-value corresponding to the pathway was computed using only over-representation analysis.
