# Supplementary material for: Optimizing Precision Medicine for Breast Cancer Brain Metastases with Functional Drug Response Assessment
Source: Cancer Res Commun. 2023 Jun 21;3(6):1093–103. doi: 10.1158/2767-9764.CRC-22-0492 (PMC10284082; doi:10.1158/2767-9764.CRC-22-0492)
Supplement: Supplementary Data S5 — GSEA analysis using the oncogenic, KEGG, and GOBP gene sets. [file crc-22-0492-s05.pdf]

S5. GSEA analysis using the oncogenic, KEGG, and GOBP gene sets.

GSEA table for Oncogenic up-regulated gene sets (FDR < 0.05)

NAME	SIZE	ES	NES	p-val	FDR q-value
PIGF_UP.V1_UP	184	0.37999806	2.1691117	0	7.66E-05
ERBB2_UP.V1_DN	190	0.35316053	2.0094836	0	0.001074609
TBK1.DF_DN	280	0.28347325	1.7019244	0	0.015042434
VEGF_A_UP.V1_DN	185	0.29627037	1.6866676	0	0.013255387
RB_P107_DN.V1_DN	120	0.3023082	1.613606	8.74E-04	0.02098995
RB_P130_DN.V1_DN	126	0.2854445	1.5376194	0.003938731	0.036148705

GSEA table for Oncogenic down-regulated gene sets (FDR < 0.05)

NAME	SIZE	ES	NES	p-val	FDR q-value
KRAS.PROSTATE_UP.V1_UP	98	-0.5134683	-2.299555	0	0
KRAS.600.LUNG.BREAST_UP.V1_UP	176	-0.4695035	-2.2835803	0	0
KRAS.BREAST_UP.V1_UP	89	-0.4960229	-2.1829803	0	0
KRAS.LUNG.BREAST_UP.V1_UP	93	-0.4846052	-2.1521356	0	0
KRAS.LUNG_UP.V1_UP	94	-0.4757321	-2.1105325	0	6.28E-05
CAHOY_NEURONAL	62	-0.4963074	-2.051618	0	7.63E-05
P53_DN.V2_UP	96	-0.4545239	-2.037276	0	9.39E-05
RELA_DN.V1_DN	97	-0.4399677	-1.9602392	0	5.10E-04
IL15_UP.V1_DN	126	-0.4122638	-1.920542	0	7.79E-04
JAK2_DN.V1_UP	108	-0.4216185	-1.9118984	1.32E-04	7.64E-04
RPS14_DN.V1_UP	169	-0.3853375	-1.8674365	0	0.00126314
LEF1_UP.V1_UP	172	-0.3777391	-1.8274897	0	0.00204783
IL21_UP.V1_UP	145	-0.3804847	-1.8082885	1.27E-04	0.00239922
PTEN_DN.V1_DN	121	-0.3888049	-1.7984138	1.30E-04	0.00248792
BRCA1_DN.V1_UP	82	-0.4115107	-1.7910712	4.09E-04	0.00261858
ATF2_UP.V1_UP	126	-0.3837714	-1.7843652	0	0.00265331
IL2_UP.V1_UP	159	-0.3689195	-1.7741044	1.26E-04	0.00290406
KRAS.KIDNEY_UP.V1_DN	88	-0.3982481	-1.749471	0.00149477	0.003836
DCA_UP.V1_UP	126	-0.3741754	-1.7391055	7.67E-04	0.00420364
KRAS.600_UP.V1_UP	203	-0.3503501	-1.7383595	0	0.00403845
KRAS.300_UP.V1_UP	107	-0.3753077	-1.7025163	6.58E-04	0.00607616
P53_DN.V1_DN	173	-0.3507869	-1.6983001	2.48E-04	0.00620596
KRAS.50_UP.V1_UP	38	-0.4575638	-1.6971632	0.00627615	0.00605075
RB_P107_DN.V1_UP	125	-0.3657492	-1.6965069	5.15E-04	0.00585956
SRC_UP.V1_UP	124	-0.3638451	-1.691479	5.16E-04	0.00602709
IL15_UP.V1_UP	161	-0.3499104	-1.6811961	6.25E-04	0.00657376
PIGF_UP.V1_DN	148	-0.3472409	-1.6576235	7.57E-04	0.00844398
KRAS.BREAST_UP.V1_DN	103	-0.3665667	-1.6526378	0.00197576	0.00869104
E2F3_UP.V1_UP	168	-0.3394752	-1.6401621	4.95E-04	0.00973901
JNK_DN.V1_DN	146	-0.3449201	-1.6313322	3.82E-04	0.01046878
CAMP_UP.V1_DN	186	-0.3264933	-1.6034255	7.39E-04	0.01410773
PTEN_DN.V1_UP	135	-0.3399975	-1.5964662	0.00254485	0.01489654
ALK_DN.V1_UP	100	-0.3515601	-1.5770893	0.00436681	0.01821641
CAHOY_ASTROGLIAL	88	-0.3584442	-1.5765339	0.00569878	0.01780232
VEGF_A_UP.V1_UP	158	-0.3253332	-1.5661472	0.00163132	0.01944393
CYCLIN_D1_UP.V1_UP	154	-0.3264754	-1.5609566	0.00302077	0.02010019
PRC1_BMI_UP.V1_UP	141	-0.3300336	-1.5605282	0.00292733	0.01969357
PDGF_UP.V1_DN	94	-0.3529673	-1.5599719	0.00720865	0.0192949
PRC2_SUZ12_UP.V1_UP	141	-0.325922	-1.5430727	0.00457724	0.02276749
RB_P130_DN.V1_UP	109	-0.3356836	-1.5277474	0.00834769	0.02628136
KRAS.AMP.LUNG_UP.V1_DN	106	-0.3370817	-1.5270163	0.00862527	0.0258224
KRAS.PROSTATE_UP.V1_DN	94	-0.3437824	-1.5264548	0.01114244	0.02538833
KRAS.600.LUNG.BREAST_UP.V1_DN	198	-0.3088274	-1.5213447	0.00302737	0.02621664
IL2_UP.V1_DN	143	-0.3190751	-1.5122533	0.00696291	0.02844425
ATF2_S_UP.V1_UP	146	-0.3157506	-1.5024015	0.00497894	0.03117847
PKCA_DN.V1_DN	120	-0.3231099	-1.4912496	0.0098129	0.0345175
JNK_DN.V1_UP	142	-0.3151889	-1.4871517	0.00818125	0.03545722
SNF5_DN.V1_UP	162	-0.3081793	-1.4846565	0.00732646	0.0357143
SNF5_DN.V1_DN	133	-0.3155903	-1.4754627	0.01210716	0.03864779
YAP1_UP	43	-0.3830668	-1.4662932	0.03778338	0.04172044
BMI1_DN_MEL18_DN.V1_UP	129	-0.3098199	-1.4555154	0.01447028	0.04582575

GSEA table for KEGG up-regulated gene sets (FDR < 0.05)

NAME	SIZE	ES	NES	p-val	FDR q-value
KEGG_PROTEIN_EXPORT	23	0.631071	2.3141265	0	9.33E-04
KEGG_OXIDATIVE_PHOSPHORYLATION	121	0.42155826	2.2039602	0	0.002276881
KEGG_PARKINSONS_DISEASE	117	0.3875401	2.0368695	0	0.0068126
KEGG_CITRATE_CYCLE_TCA_CYCLE	29	0.527474	2.056116	0	0.007522518

GSEA table for KEGG down-regulated gene sets (FDR < 0.05)

NAME	SIZE	ES	NES	p-val	FDR q-value
KEGG_GRAFT_VERSUS_HOST_DISEASE	26	-0.84030366	-2.515704	0	0
KEGG_AUTOIMMUNE_THYROID_DISEASE	25	-0.8427207	-2.5131416	0	0
KEGG_INTESTINAL_IMMUNE_NETWORK_FOR_IGA_PRODUCTION	32	-0.80258304	-2.5083468	0	0
KEGG_HEMATOPOIETIC_CELL_LINEAGE	62	-0.71402407	-2.4952674	0	0
KEGG_ALLOGRAFT_REJECTION	26	-0.82865447	-2.4935515	0	0
KEGG_ASTHMA	17	-0.9046613	-2.4577773	0	0
KEGG_PRIMARY_IMMUNODEFICIENCY	26	-0.75680894	-2.2673798	0	0
KEGG_CYTOKINE_CYTOKINE_RECEPTOR_INTERACTION	159	-0.585377	-2.2642326	0	0
KEGG_TYPE_I_DIABETES_MELLITUS	31	-0.7248778	-2.2580779	0	0
KEGG_LEISHMANIA_INFECTION	61	-0.6452481	-2.2441857	0	0
KEGG_CELL_ADHESION_MOLECULES_CAMS	112	-0.5878192	-2.2034686	0	0
KEGG_VIRAL_MYOCARDITIS	60	-0.6162249	-2.1354454	0	0
KEGG_ECM_RECEPTOR_INTERACTION	81	-0.56642216	-2.05313	0	5.46E-05
KEGG_NEUROACTIVE_LIGAND_RECEPTOR_INTERACTION	147	-0.5096956	-1.9569695	0	2.93E-04
KEGG_SYSTEMIC_LUPUS_ERYTHEMATOSUS	103	-0.50952756	-1.898131	0	8.67E-04
KEGG_CALCIUM_SIGNALING_PATHWAY	143	-0.48741472	-1.8675388	0	0.001418914
KEGG_ANTIGEN_PROCESSING_AND_PRESENTATION	54	-0.5400128	-1.8550544	0	0.001746663
KEGG_CHEMOKINE_SIGNALING_PATHWAY	142	-0.45718867	-1.7563272	0	0.006958953
KEGG_NATURAL_KILLER_CELL_MEDIATED_CYTOTOXICITY	93	-0.46455982	-1.717646	1.07E-04	0.011132123
KEGG_DRUG_METABOLISM_CYTOCHROME_P450	31	-0.54923296	-1.7078425	0.003699593	0.01209183
KEGG_DILATED_CARDIOMYOPATHY	74	-0.46650556	-1.6775744	8.86E-04	0.01647371
KEGG_FOCAL_ADHESION	190	-0.42584896	-1.6657133	0	0.018245969
KEGG_ABC_TRANSPORTERS	39	-0.5074533	-1.6487919	0.005799503	0.021292005
KEGG_LEUKOCYTE_TRANSENDOTHELIAL_MIGRATION	97	-0.44282904	-1.6397674	0.001071467	0.022794547
KEGG_HYPERTROPHIC_CARDIOMYOPATHY_HCM	67	-0.46614856	-1.6393937	0.002217541	0.021970354
KEGG_T_CELL_RECEPTOR_SIGNALING_PATHWAY	93	-0.44438422	-1.6342639	0.001503114	0.022440786
KEGG_B_CELL_RECEPTOR_SIGNALING_PATHWAY	71	-0.45687383	-1.6306418	0.001659476	0.022519669
KEGG_JAK_STAT_SIGNALING_PATHWAY	99	-0.43428278	-1.6104509	0.001070893	0.027168183
KEGG_COMPLEMENT_AND_COAGULATION_CASCADES	51	-0.4630738	-1.5760622	0.009388039	0.037909135

GSEA table for GOBP up-regulated gene sets (FDR < 0.05)

NAME	SIZE	ES	NES	p-val	FDR q-value
GOBP_MITOCHONDRIAL_TRANSLATIONAL_TERMINATION	89	0.5148428	2.5802567	0	7.97E-04
GOBP_ER_NUCLEUS_SIGNALING_PATHWAY	52	0.55574876	2.5132298	0	7.99E-04
GOBP_ATP_SYNTHESIS_COUPLED_ELECTRON_TRANSPORT	93	0.47469223	2.4081469	0	0.00300918
GOBP_REGULATION_OF_TRANSCRIPTION_FROM_RNA_POLYMERASE_II_PROMOTER_IN_RESPONSE_TO_HYPOXIA	74	0.48969692	2.375456	0	0.00391304
GOBP_TRANSLATIONAL_TERMINATION	104	0.44788104	2.320398	0	0.00644285
GOBP_OXIDATIVE_PHOSPHORYLATION	135	0.42839652	2.2987373	0	0.00667296
GOBP_REGULATION_OF_DNA_TEMPLATED_TRANSCRIPTION_IN_RESPONSE_TO_STRESS	109	0.42872593	2.2605312	0	0.00908123
GOBP_VIRION_ASSEMBLY	40	0.5290905	2.2503767	0	0.00885648
GOBP_AEROBIC_RESPIRATION	81	0.44912013	2.2193592	0	0.01118527
GOBP_CELLULAR_RESPONSE_TO_TOPOLOGICALLY_INCORRECT_PROTEIN	156	0.39882573	2.21625	0	0.01042456
GOBP_MITOCHONDRIAL_ELECTRON_TRANSPORT_CYTOCHROME_C_TO_OXYGEN	18	0.65130705	2.215675	0	0.00958378
GOBP_RESPIRATORY_ELECTRON_TRANSPORT_CHAIN	108	0.42451945	2.2020218	0	0.01069079
GOBP_MITOCHONDRIAL_TRANSLATION	134	0.40634346	2.183095	0	0.01220119
GOBP_POSITIVE_REGULATION_OF_DNA_TEMPLATED_TRANSCRIPTION_ELONGATION	25	0.580468	2.1814685	0	0.01154373
GOBP_REGULATION_OF_NUCLEOBASE_CONTAINING_COMPOUND_TRANSPORT	15	0.6850738	2.180666	7.55E-04	0.0108532
GOBP_ENDOPLASMIC_RETICULUM_MANNOSE_TRIMMING	16	0.66398025	2.1683912	7.80E-04	0.01178939
GOBP_GOLGI_ORGANIZATION	127	0.40118575	2.1625478	0	0.01179032
GOBP_REGULATION_OF_ENDOPLASMIC_RETICULUM_UNFOLDED_PROTEIN_RESPONSE	28	0.54766536	2.127553	4.99E-04	0.01662011
GOBP_TRANSCRIPTION_ELONGATION_FROM_RNA_POLYMERASE_II_PROMOTER	81	0.42699003	2.106973	0	0.019527
GOBP_ANAPHASE_PROMOTING_COMPLEX_DEPENDENT_CATABOLIC_PROCESS	78	0.4278212	2.1052907	0	0.01881966
GOBP_COPII_COATED_VESICLE_BUDDING	71	0.43600196	2.1025982	0	0.01860879
GOBP_VESICLE_CARGO_LOADING	20	0.5994502	2.0997443	4.23E-04	0.01841475
GOBP_RESPONSE_TO_TOPOLOGICALLY_INCORRECT_PROTEIN	191	0.37317285	2.0983012	0	0.01785151
GOBP_UBIQUITIN_DEPENDENT_PROTEIN_CATABOLIC_PROCESS_VIA_THE_MULTIVESICULAR_BODY_SORTING_PATHWAY	23	0.56153846	2.0708737	0	0.02233192
GOBP_ENDOPLASMIC_RETICULUM_UNFOLDED_PROTEIN_RESPONSE	117	0.3951727	2.0703301	0	0.02149398
GOBP_MITOCHONDRIAL_GENE_EXPRESSION	165	0.37187043	2.069239	0	0.02093118
GOBP_INTRA_GOLGI_VESICLE_MEDIATED_TRANSPORT	30	0.52455705	2.0665505	0.00105152	0.02068525
GOBP_INTEGRATED_STRESS_RESPONSE_SIGNALING	26	0.54361326	2.0619016	4.93E-04	0.02084304
GOBP_POSITIVE_REGULATION_OF_HISTONE_DEACETYLATION	17	0.61732966	2.0602777	0.00118017	0.0203892
GOBP_RNA_CAPPING	33	0.50926226	2.0590794	0	0.02004727
GOBP_ENDOPLASMIC_RETICULUM_TO_GOLGI_VESICLE_MEDIATED_TRANSPORT	189	0.36615774	2.053904	0	0.02038059
GOBP_CELLULAR_RESPONSE_TO_STEROL_DEPLETION	16	0.61905855	2.0474834	0.00113982	0.02082402
GOBP_TRANSLATIONAL_ELONGATION	131	0.37939736	2.0455341	0	0.02055887
GOBP_RNA_EXPORT_FROM_NUCLEUS	130	0.38322088	2.0451307	0	0.02003722
GOBP_STEROL_BIOSYNTHETIC_PROCESS	66	0.4281963	2.0429168	0	0.0198856
GOBP_VESICLE_BUDDING_FROM_MEMBRANE	107	0.3941925	2.0427673	0	0.01934462
GOBP_PROTEIN_QUALITY_CONTROL_FOR_MISFOLDED_OR_INCOMPLETELY_SYNTHESIZED_PROTEINS	28	0.52611256	2.0413876	0	0.01907022
GOBP_VESICLE_TARGETING_TO_FROM_OR_WITHIN_GOLGI	72	0.41854268	2.0374014	0	0.0192738
GOBP_MRNA_EXPORT_FROM_NUCLEUS	105	0.39030963	2.024657	0	0.02098905
GOBP_POSITIVE_REGULATION_OF_PROTEIN_DEACETYLATION	21	0.56271714	2.0165682	0.00173989	0.02218876
GOBP_DNA_TEMPLATED_TRANSCRIPTION_ELONGATION	110	0.38521206	2.009029	0.0018018	0.02305034
GOBP_POSITIVE_REGULATION_OF_CHROMOSOME_SEPARATION	17	0.59824973	2.0012763	0.00119379	0.0239352
GOBP_RETROGRADE_VESICLE_MEDIATED_TRANSPORT_GOLGI_TO_ENDOPLASMIC_RETICULUM	83	0.40226033	2.001097	0	0.02341957
GOBP_REGULATION_OF_CHOLESTEROL_BIOSYNTHETIC_PROCESS	42	0.46410406	2.0006864	6.29E-04	0.02294799
GOBP_UBIQUITIN_DEPENDENT_ERAD_PATHWAY	76	0.40638992	1.9988033	0	0.02279347
GOBP_MITOCHONDRIAL_ELECTRON_TRANSPORT_NADH_TO_UBIQUINONE	52	0.440862	1.9870356	0	0.02451197
GOBP_TRICARBOXYLIC_ACID_CYCLE	33	0.48046908	1.9466982	0.00164294	0.03401632
GOBP_CHAPERONE_MEDIATED_AUTOPHAGY	16	0.5866304	1.9298283	0.00195389	0.03828349
GOBP_NEGATIVE_REGULATION_OF_MACROAUTOPHAGY	35	0.47340983	1.9274449	5.67E-04	0.03824441
GOBP_INNER_MITOCHONDRIAL_MEMBRANE_ORGANIZATION	56	0.41934046	1.9268379	0	0.03772754
GOBP_PHOSPHATIDYLCHOLINE_BIOSYNTHETIC_PROCESS	35	0.46212283	1.8968313	0.00235849	0.04686664

GSEA table for GOBP down-regulated gene sets (FDR < 0.05)

NAME	SIZE	ES	NES	p-val	FDR q-value
GOBP_B_CELL_RECEPTOR_SIGNALING_PATHWAY	64	-0.7669317	-2.6880314	0	0
GOBP_ADAPTIVE_IMMUNE_RESPONSE	342	-0.6237414	-2.5076084	0	0
GOBP_ALPHA_BETA_T_CELL_ACTIVATION	121	-0.6303483	-2.387556	0	0
GOBP_POSITIVE_REGULATION_OF_CELL_ACTIVATION	289	-0.596866	-2.3836842	0	0
GOBP_POSITIVE_REGULATION_OF_T_CELL_PROLIFERATION	77	-0.6579624	-2.375067	0	0
GOBP_POSITIVE_REGULATION_OF_LEUKOCYTE_PROLIFERATION	117	-0.6276081	-2.360609	0	0
GOBP_B_CELL_ACTIVATION	219	-0.5969783	-2.3569849	0	0
GOBP_POSITIVE_REGULATION_OF_LEUKOCYTE_CELL_CELL_ADHESION	196	-0.6006042	-2.3548284	0	0
GOBP_REGULATION_OF_B_CELL_ACTIVATION	115	-0.6243017	-2.3476071	0	0
GOBP_ALPHA_BETA_T_CELL_DIFFERENTIATION	89	-0.6387314	-2.3447883	0	0
GOBP_POSITIVE_REGULATION_OF_B_CELL_ACTIVATION	77	-0.6481271	-2.3360827	0	0
GOBP_B_CELL_PROLIFERATION	67	-0.6596063	-2.3290925	0	0
GOBP_REGULATION_OF_T_CELL_DIFFERENTIATION	123	-0.6105623	-2.3231664	0	0
GOBP_REGULATION_OF_LEUKOCYTE_PROLIFERATION	187	-0.5929681	-2.319425	0	0
GOBP_T_CELL_DIFFERENTIATION	206	-0.5874825	-2.3170707	0	0
GOBP_REGULATION_OF_LYMPHOCYTE_DIFFERENTIATION	148	-0.5985021	-2.311673	0	0
GOBP_CELLULAR_DEFENSE_RESPONSE	38	-0.7124783	-2.3060017	0	0
GOBP_REGULATION_OF_LYMPHOCYTE_ACTIVATION	370	-0.5709478	-2.3055615	0	0
GOBP_LYMPHOCYTE_MIGRATION	80	-0.6339475	-2.30338	0	0
GOBP_T_CELL_SELECTION	38	-0.7085554	-2.297034	0	0
GOBP_LEUKOCYTE_PROLIFERATION	238	-0.5752217	-2.28417	0	0
GOBP_ANTIGEN_RECEPTOR_MEDIATED_SIGNALING_PATHWAY	233	-0.5761899	-2.2796462	0	0
GOBP_REGULATION_OF_T_CELL_ACTIVATION	265	-0.5705994	-2.2735913	0	0
GOBP_REGULATION_OF_ALPHA_BETA_T_CELL_ACTIVATION	80	-0.6269385	-2.271247	0	0
GOBP_POSITIVE_REGULATION_OF_CELL_CELL_ADHESION	230	-0.5724195	-2.26788	0	0
GOBP_T_CELL_MIGRATION	53	-0.6656491	-2.2654603	0	0
GOBP_INTERLEUKIN_4_PRODUCTION	29	-0.7378844	-2.2573328	0	0
GOBP_ADAPTIVE_IMMUNE_RESPONSE_BASED_ON_SOMATIC_RECOMBINATION_OF_IMMUNE_RECEPTORS_BUILT_FROM_IMMUNOGLOBULIN_SUPERFAMILY_DOMAINS	234	-0.5687107	-2.2492518	0	2.98E-06
GOBP_REGULATION_OF_CELL_ACTIVATION	468	-0.5520781	-2.245426	0	2.88E-06
GOBP_MONONUCLEAR_CELL_DIFFERENTIATION	336	-0.5564137	-2.2389948	0	2.78E-06
GOBP_POSITIVE_REGULATION_OF_ALPHA_BETA_T_CELL_ACTIVATION	55	-0.6518996	-2.2327092	0	2.69E-06
GOBP_HUMORAL_IMMUNE_RESPONSE_MEDIATED_BY_CIRCULATING_IMMUNOGLOBULIN	56	-0.6452255	-2.2216325	0	2.61E-06
GOBP_LYMPHOCYTE_MEDIATED_IMMUNITY	213	-0.5613896	-2.2115097	0	7.56E-06
GOBP_INTERFERON_GAMMA_PRODUCTION	86	-0.6052293	-2.2098887	0	7.34E-06
GOBP_POSITIVE_REGULATION_OF_LYMPHOCYTE_DIFFERENTIATION	88	-0.601038	-2.200775	0	1.43E-05
GOBP_NEGATIVE_REGULATION_OF_INTERLEUKIN_6_PRODUCTION	36	-0.6867633	-2.1974807	0	1.85E-05
GOBP_T_CELL_PROLIFERATION	152	-0.5719871	-2.1970255	0	1.80E-05
GOBP_T_CELL_ACTIVATION	380	-0.5405189	-2.183739	0	1.97E-05
GOBP_REGULATION_OF_ANTIGEN_RECEPTOR_MEDIATED_SIGNALING_PATHWAY	58	-0.6304706	-2.182941	0	1.92E-05
GOBP_RESPONSE_TO_CHEMOKINE	57	-0.6328475	-2.1828885	0	1.87E-05
GOBP_INTERLEUKIN_10_PRODUCTION	46	-0.6526682	-2.174941	0	2.03E-05
GOBP_REGULATION_OF_B_CELL_PROLIFERATION	52	-0.6369289	-2.174079	0	1.98E-05
GOBP_LEUKOCYTE_CELL_CELL_ADHESION	291	-0.5428124	-2.172444	0	1.94E-05
GOBP_REGULATION_OF_B_CELL_RECEPTOR_SIGNALING_PATHWAY	23	-0.744792	-2.1714225	0	2.08E-05
GOBP_CD4_POSITIVE_ALPHA_BETA_T_CELL_DIFFERENTIATION	66	-0.6157419	-2.1699202	0	2.04E-05
GOBP_INTERLEUKIN_2_PRODUCTION	55	-0.632943	-2.167681	0	2.17E-05
GOBP_POSITIVE_REGULATION_OF_ANTIGEN_RECEPTOR_MEDIATED_SIGNALING_PATHWAY	21	-0.7550752	-2.1667323	0	2.13E-05
GOBP_MONONUCLEAR_CELL_MIGRATION	140	-0.5624852	-2.1527915	0	2.43E-05
GOBP_LEUKOCYTE_TETHERING_OR_ROLLING	25	-0.7235997	-2.1509452	0	2.72E-05
