# Supplementary material for: Optimizing Precision Medicine for Breast Cancer Brain Metastases with Functional Drug Response Assessment
Source: Cancer Res Commun. 2023 Jun 21;3(6):1093–103. doi: 10.1158/2767-9764.CRC-22-0492 (PMC10284082; doi:10.1158/2767-9764.CRC-22-0492)
Supplement: Supplementary Data S6 — Clinicopathologic characteristics of source patients for brain metastases PDX models: Group B. [file crc-22-0492-s06.pdf]

S6. Clinicopathologic characteristics of source patients for brain metastases PDX models: Group B.

PDX ID	Age at BC	Age at BrMet	Germline mutation	Clinical subtype	Prior CNS directed therapy exposure	Prior systemic therapy exposure	Non-CNS disease sites	OS from BrMet in years
BC3	32	34	BRCA1	TNBC	None	Adjuvant CT (AC-T) – possibly Olaparib*	Bone, lung, LN	0.9
BC4	51	57	None	HR+ HER2-	None	Adjuvant CT (AC-T), adjuvant AI -> metastatic: FUL, AI+ PAL, capecitabine, vinorelbine	Lung	Alive** (4.2years)
BC6	55	69	None	HER2+	None	Adjuvant CT (TAC)-> metastatic: capecitabine, tamoxifen, FUL, docetaxel/HP, AI/lapatinib, T-DM1	Lung, pleura, LN, bone	1.3
BC9	26	27	RAD50	TNBC	None	Adjuvant CT (AC-T)	Lung, bone, pericardium, LM	0.6
BC11	66	71	None	HR+ HER2-	None	Adjuvant CT (AC-T), adjuvant tamoxifen -> metastatic: AI+PAL, FUL+EVE, capecitabine, eribulin, vinorelbine	Lung, pleura, skin, soft tissue, bone.	0.3
BC16	43	44	None	HER2+	SRS	Neoadjuvant CT (AC-TH)	None	Alive** (5.4years)

* Unknown if received olaprib or placebo on a clinical trial
** Alive at the last follow up time noted

BC, breast cancer, BrMet, brain metastases, CNS, central nervous system, OS, overall survival from the brain metastases diagnosis, TNBC, triple negative breast cancer, HR, hormonal receptor, HER2, human epidermal growth factor 2, SRS, stereotactic radiosurgery, CT, chemotherapy, AC-T, doxorubicin and cyclophosphamide followed by paclitaxel, TAC, docetaxel, doxorubicin, and cyclophosphamide, AI, aromatase inhibitor, HP, trastuzumab and Pertuzumab, T-DM1, ado-trastuzumab emtansine, AC-TH, doxorubicin and cyclophosphamide followed by paclitaxel and trastuzumab, LN, lymph nodes, LM, leptomeningeal metastases.
