# Supplementary material for: Optimizing Precision Medicine for Breast Cancer Brain Metastases with Functional Drug Response Assessment
Source: Cancer Res Commun. 2023 Jun 21;3(6):1093–103. doi: 10.1158/2767-9764.CRC-22-0492 (PMC10284082; doi:10.1158/2767-9764.CRC-22-0492)
Supplement: Supplementary Data S8 — DSS3 values for chemotherapies commonly used in breast cancer treatment [file crc-22-0492-s08.pdf]

S8. DSS_3 values for chemotherapies commonly used in breast cancer treatment

Drug	BC3	BC9	BC4	BC11	BC6	BC16
Doxorubicin	12	8	16	1	13	4
Docetaxel	2	31	0	0	0	0
Paclitaxel	0	13	18	2	16	0
Eribulin Mesylate	52	8	60	0	41	46
5-Fluorouracil (prodrug capecitabine)	2	2	11	0	2	2
Carboplatin	0	3	0	0	1	0
Gemcitabine	62	37	0	0	0	0
SN-38 (payload of sacituzumab)	49	54	14	6	9	10

Prior exposure in red
